# Serum Levels of Exocrine Pancreatic Enzymes in Patients with Acute Decompensated Heart Failure

**DOI:** 10.31083/RCM28160

**Published:** 2025-03-19

**Authors:** Masaru Hiki, Takatoshi Kasai, Akihiro Sato, Sayaki Ishiwata, Shoichiro Yatsu, Jun Shitara, Hiroki Matsumoto, Megumi Shimizu, Azusa Murata, Takao Kato, Shoko Suda, Hiroyuki Daida

**Affiliations:** ^1^Department of Cardiovascular Biology and Medicine, Juntendo University School of Medicine, 113-8421 Tokyo, Japan; ^2^Cardiovascular Respiratory Sleep Medicine, Juntendo University Graduate School of Medicine, 113-8421 Tokyo, Japan; ^3^Department of Cardiovascular Management and Remote Monitoring, Juntendo University Graduate School of Medicine, 113-8421 Tokyo, Japan; ^4^Sleep and Sleep Disordered Breathing Center, Juntendo University Hospital, 113-8421 Tokyo, Japan; ^5^Department of Cardiology, Juntendo University Shizuoka Hospital, 410-2295 Shizuoka, Japan

**Keywords:** amylase, congestion, hypoperfusion, lipase, malnutrition

## Abstract

**Background:**

Interactions between the heart and other organs have been a focus in acute decompensated heart failure (ADHF). However, the association between ADHF and pancreatic exocrine insufficiency (PEI), which may lead to malnutrition, remains unclear. We investigated the relationship between exocrine pancreatic enzymes and ADHF.

**Methods:**

We enrolled 155 and 46 patients with and without ADHF, respectively. Serum amylase and lipase levels were compared between the two groups. In the ADHF group, factors correlating with serum amylase or lipase levels were assessed using multiple regression analysis, and changes in their levels throughout the hospital course were determined.

**Results:**

Patients with ADHF exhibited significantly lower amylase and lipase levels. In the same group, the significant independent correlates of lower amylase levels included a lower blood urea–nitrogen level (partial correlation coefficient, 0.530; *p* < 0.001), lower albumin level (partial correlation coefficient, 0.252; *p* = 0.015), and higher uric acid level (partial correlation coefficient, –0.371; *p* < 0.001). The significant independent correlates of lower lipase levels included coexisting atrial fibrillation (coefficient, 0.287; *p* = 0.026), lower creatinine level (coefficient, 0.236; *p* = 0.042), and higher B-type natriuretic peptide level (coefficient, –0.257; *p* = 0.013). Both amylase and lipase levels significantly increased following the improvement in ADHF.

**Conclusions:**

In patients with ADHF, decreased serum amylase and lipase levels were associated with the congestion severity, suggesting that PEI may occur in patients with ADHF, potentially due to ADHF-related congestion.

## 1. Introduction

Despite recent advances in treatment options, heart failure (HF) remains a significant public health concern, partly due to poor nutritional status. Patients with HF are often reported to have poor nutritional status [1, 2], and coexisting poor nutritional status is associated with worse clinical outcomes in patients with stable and acute decompensated HF (ADHF) [3, 4, 5, 6].

The pathophysiology of HF, involving congestion and/or hypoperfusion, leads to organ damage, affecting organs such as kidneys [7], lungs [8], and liver [9]. The pancreas may also be one of the affected organs. Several reports suggested that hypoperfusion associated with HF might cause pancreatic damage [10, 11, 12, 13] and increase the serum levels of pancreatic exocrine enzymes, such as amylase and lipase. A 1925 study reported that chronic passive congestion associated with ADHF could induce atrophy of the pancreatic acinar cells and lead to the disappearance of prezymogen granules within these atrophied pancreatic acinar cells [14]. These changes may result in the decreased production and release of pancreatic exocrine enzymes. Furthermore, although the interval from the first episode of ADHF to death may not have a significant effect—despite the occurrence of repeated ADHF episodes during this period—a longer duration of congestion during the recent ADHF episode has been associated with more advanced changes in pancreatic tissues [14]. These findings suggest that active congestion related to ADHF may alter pancreatic tissue morphology and potentially impact exocrine function. However, these effects on the pancreas may be reversible and depend on the presence or absence of ongoing congestion. Despite this, little attention has been paid to alterations in pancreatic tissues and the potential for pancreatic exocrine insufficiency (PEI) in patients with HF. As pancreatic exocrine enzymes are essential for nutrient digestion, a decrease in the release of pancreatic exocrine enzymes can lead to maldigestion, resulting in malabsorption of fat, protein, and fat-soluble vitamins, ultimately contributing to poor nutritional status [15, 16]. This may be particularly true for patients with HF. However, specific data focusing on PEI in patients with HF are lacking.

Therefore, we aimed to test the hypothesis that serum levels of exocrine pancreatic enzymes, including amylase and lipase, which are indicative of pancreatic exocrine function [17], may be lower in patients with ADHF compared with those without HF. It was also hypothesized that the low serum levels of these enzymes are associated with parameters of nutritional status and congestion in hospitalized patients with ADHF, and that changes in the serum levels of exocrine pancreatic enzymes may correspond to improvements in HF.

## 2. Materials and Methods

### 2.1 Study Participants

We enrolled 155 consecutive patients who were admitted to Juntendo University Hospital, Tokyo, Japan, with a diagnosis of ADHF from November 2014 to December 2015. Additionally, 46 patients without a history or symptoms of HF were assigned as the control group. A diagnosis of ADHF requiring hospitalization was made based on the modified Framingham criteria [18]. The control group comprised patients admitted for coronary angiogram and/or elective percutaneous coronary intervention and those admitted for implantable device generator replacement or elective ablation procedures. Patients undergoing dialysis and those with neoplasms were excluded. This study was approved by the Institutional Review Board of Juntendo University Hospital (#871) and was conducted in accordance with the Declaration of Helsinki. Informed consent was obtained from all patients.

### 2.2 Data Collection

All clinical data at the initial presentation were prospectively collected. Serum amylase levels, lipase levels, and other baseline data were obtained within the first 24 h after admission. A complete two-dimensional echocardiography was performed in each patient, with the left ventricular ejection fraction (LVEF) calculated using the modified Simpson method. Smoking habit was defined as current smoking or smoking cessation in <1 year prior to admission. Habitual drinking was defined as consuming more than 20 g of alcohol per day at least three times a week.

### 2.3 Study Protocol

This three-part study was conducted to evaluate relationships between serum levels of exocrine pancreatic enzymes, as suggestive of pancreatic exocrine function and ADHF.

In study 1, the serum levels of exocrine pancreatic enzymes, including serum amylase and lipase, were compared between patients with ADHF and controls. To reduce potential bias caused by imbalances in baseline characteristics, matching was carried out based on age, sex, and body mass index (BMI). A 2:1 matching ratio (two patients with ADHF for each control participant) was employed to maximize statistical power. The matching criteria used were age within ± 3.0 years and BMI within ± 3.0 kg/m^2^. Matching was performed by an investigator who was blinded to all patients’ characteristics, except for age, sex, and BMI.

In study 2, using the prematched patients with ADHF, the factors associated with the serum levels of amylase and lipase were identified using univariable and multivariable regression analyses.

In study 3, the changes in the serum levels of amylase and lipase throughout the hospitalization period (i.e., on admission and a few days before discharge) were determined in a subset of prematched patients with ADHF. Additionally, changes in body weight, hemoglobin, blood urea nitrogen (BUN), serum albumin, creatinine, sodium, potassium, and plasma B-type natriuretic peptide (BNP) levels from admission to discharge were assessed.

### 2.4 Statistical Analysis

Continuous variables were expressed as the mean ± standard deviation for normally distributed data and median (interquartile range) for non-normally distributed data. Categorical variables were reported as numbers and percentages. To compare the baseline characteristics between the ADHF group and control group, χ^2^ test or Fisher’s exact test was used for categorical variables, whereas *t*-test or Mann–Whitney U-test was used for continuous variables. In study 2, among prematched patients with ADHF, multivariable stepwise linear regression analyses (with *p* < 0.05 as an inclusion criterion and *p* > 0.1 as an exclusion criterion) were performed using natural logarithm-transformed serum levels of amylase or lipase (Log-amylase and Log-lipase, respectively) as a dependent variable in addition to the following independent variables: age; sex; BMI; systolic and diastolic blood pressures (BP); heart rate; history of HF; ischemic etiology; atrial fibrillation (AF); LVEF; hemoglobin, cholinesterase, albumin, BUN, creatinine, uric acid, triglyceride, low density lipoprotein (LDL) cholesterol, high density lipoprotein (HDL) cholesterol, glucose, and hemoglobin A1c (HbA1c) levels; serum levels of sodium and potassium; natural logarithm transformed of C-reactive protein (Log-CRP); natural logarithm transformed BNP (Log-BNP); and medication used at admission. Independent variables with a *p* value of <0.05 in the univariable analyses were subsequently included in the multivariable analysis. In study 3, Log-amylase, Log-lipase in addition to body weight, hemoglobin, BUN, albumin, creatinine, sodium, potassium, and Log-BNP levels were compared between admission and predischarge using a *t*-test. The correlations between changes in amylase or lipase levels and other parameters were assessed using Pearson’s correlation coefficient. A *p* value of less than 0.05 was considered significant. All analyses were performed using SPSS version 23.0 (IBM Corp., Armonk, NY, USA).

## 3. Results

In the ADHF group, patients with end-stage renal disease undergoing chronic hemodialysis (n = 5) and life-threatening malignancy (n = 36) were excluded. In the control group, patients with end-stage renal disease undergoing chronic hemodialysis (n = 8) and life-threatening malignancy (n = 6) were excluded. Consequently, 114 and 32 patients with and without ADHF, respectively, were evaluated. The causes of hospitalization in the control group were as follows: 29 patients were admitted for coronary angiogram and/or elective percutaneous coronary intervention, whereas 3 patients were admitted for generator replacement or elective ablation procedures.

**Fig. 1.**
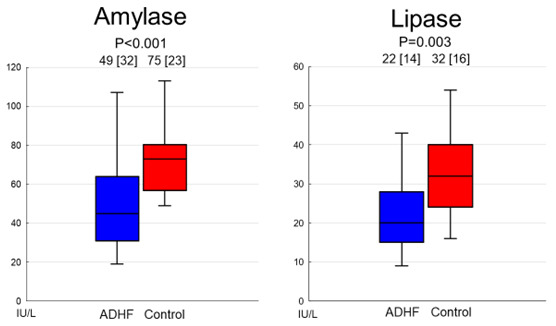
**Comparison of serum levels of amylase and lipase between patients with and without ADHF**. Both serum levels of amylase and lipase were significantly lower in patients with ADHF. Abbreviation: ADHF, acute decompensated heart failure.

In study 1, 40 patients with ADHF and 20 patients without ADHF were selected following the matching procedure. The characteristics of these postmatched patients are summarized in Table 1. Patients with ADHF were more likely to have coronary artery disease, use diuretics, and present with a higher heart rate and lower left ventricular ejection fraction. In addition, patients with ADHF had significantly lower levels of cholinesterase, albumin, and HDL cholesterol and markedly higher levels of BUN, uric acid, LDL cholesterol, BNP, and CRP compared with the control group. Patients with ADHF had significantly lower serum levels of amylase (median [interquartile range]: 49.7 [32.2] IU/L versus 75.5 [23.5] IU/L, *p* < 0.001) and lipase (22.6 [14.5] IU/L versus 32.3 [16.0] IU/L, *p* = 0.003) compared with the control group (Fig. 1).

**Table 1.** **Baseline characteristics of the matched patients**.

	ADHF	Non ADHF	*p* value
	(N = 40)	(N = 20)	
Age, years	68.8 ± 10.0	68.3 ± 10.5	0.858
Male, n (%)	32.0 (80.0)	16.0 (80.0)	1.000
Body mass index, kg/m^2^	22.7 ± 2.8	23.0 ± 2.7	0.726
Systolic blood pressure, mmHg	132.0 ± 31.9	130.8 ± 29.1	0.891
Diastolic blood pressure, mmHg	75.8 ± 19.2	73.3 ± 20.9	0.648
Heart rate, /min	87.7 ± 22.4	68.2 ± 12.6	<0.001
Left ventricular ejection fraction, %	36.7 ± 14.7	63.5 ± 11.5	<0.001
Current smoking, n (%)	9.0 (22.5)	4.0 (20.0)	0.824
Habitual drinking, n (%)	9.0 (22.5)	5.0 (25.0)	0.829
Coronary artery disease, n (%)	19.0 (47.5)	15.0 (75.0)	0.043
AF, n (%)	15.0 (37.5)	5.0 (25.0)	0.333
Hemoglobin, g/dL	12.0 ± 2.4	13.1 ± 1.7	0.083
Cholinesterase, IU/L	195.8 ± 58.3	296.8 ± 57.1	<0.001
Albumin, g/dL	3.11 ± 0.45	3.71 ± 0.45	<0.001
BUN, mg/dL	24.5 ± 12.3	16.7 ± 9.5	0.016
Creatinine, mg/dL	1.26 ± 0.76	0.95 ± 0.83	0.148
Uric acid, mg/dL	7.6 ± 2.3	5.4 ± 1.2	<0.001
Triglyceride, mg/dL	73.5 ± 22.9	106.4 ± 77.4	0.078
LDL cholesterol, mg/dL	99.6 ± 34.5	83.1 ± 19.0	0.020
HDL cholesterol, mg/dL	39.0 ± 15.6	49.8 ± 17.1	0.018
Blood glucose, mg/dL	113.6 ± 40.1	101.5 ± 29.3	0.237
HbA1c, %	6.3 ± 1.1	6.1 ± 0.9	0.491
Sodium, mmol/L	139.4 ± 3.9	140.4 ± 2.2	0.365
Potassium, mmol/L	4.0 ± 0.4	4.2 ± 0.4	0.078
BNP, pg/mL	822 (1084)	43.2 (73.0)	0.001
CRP, mg/dL	0.75 (2.97)	0.20 (0.59)	<0.001
ACE-I/ARB, n (%)	22.0 (55.0)	13.0 (65.0)	0.459
β-blockers, n (%)	20.0 (50.0)	7.0 (35.0)	0.271
MR antagonists, n (%)	7.0 (17.5)	0 (0)	0.084
Diuretics, n (%)	22.0 (55.0)	1.0 (5.0)	<0.001

Continuous variables are expressed as the mean ± standard deviation or median (interquartile range), while categorical variables are expressed as numbers (%). Abbreviations: ACE-I, angiotensin-converting enzyme inhibitor; AF, atrial fibrillation; ARB, angiotensin II receptor blocker; BNP, B-type natriuretic peptide; BUN, blood urea nitrogen; CRP, C-reactive protein; HbA1c, hemoglobin A1c; HDL, high-density lipoprotein; LDL, low-density lipoprotein; MR, mineralcorticoid receptor.

In study 2, the characteristics of prematched patients with ADHF (N = 114) are summarized in Table 2. A significant correlation was found between serum amylase and serum lipase levels (correlation coefficient, 0.646; *p* < 0.001). Multivariable stepwise linear regression analysis of patients with ADHF showed that lower BUN, lower albumin level, and higher uric acid levels were significant independent correlates of lower amylase levels, whereas the presence of AF, higher creatinine levels, and higher BNP levels were significant independent correlates of lower lipase levels (Table 3).

**Table 2.** **Baseline characteristics of all patients with ADHF**.

N = 114		
Age, years		73.3 ± 13.1
Male, n (%)		73.0 (64.0)
Body mass index, kg/m^2^		23.1 ± 4.7
Systolic blood pressure, mmHg		131.6 ± 30.7
Diastolic blood pressure, mmHg		75.9 ± 19.5
Heart rate, /min		88.2 ± 23.7
Ischemic etiology, n (%)		55.0 (48.2)
Left ventricular ejection fraction, %		41.8 ± 16.0
Nohria-Stevenson classification, n (%)		
	Subset B	90.0 (79.0)
	Subset C	20.0 (17.5)
	Subset L	4.0 (3.5)
Smoking, n (%)		17.0 (14.9)
Habitual drinking, n (%)		19.0 (16.6)
AF, n (%)		60.0 (52.6)
History of heart failure, n (%)		70.0 (61.4)
Hemoglobin, g/dL		11.6 ± 2.2
Cholinesterase, IU/L		2023.0 ± 104.7
Albumin, g/dL		3.0 ± 0.4
BUN, mg/dL		28.8 ± 16.9
Creatinine, mg/dL		1.35 ± 0.81
Uric acid, mg/dL		7.3 ± 2.3
LDL cholesterol, mg/dL		96.7 ± 36.1
HDL cholesterol, mg/dL		42.1 ± 22.4
Blood glucose, mg/dL		116.0 ± 56.2
HbA1c, %		6.2 ± 1.0
Sodium, mmol/L		139.2 ± 4.1
Potassium, mmol/L		4.0 ± 0.6
BNP, pg/mL		788 (1027)
CRP, mg/dL		0.8 (3.557)
ACE-I/ARB, n (%)		59.0 (51.8)
MR antagonists, n (%)		22.0 (19.3)
Β-blockers, n (%)		57.0 (50.0)
Diuretics, n (%)		69.0 (60.5)

Continuous variables are expressed as the mean ± standard deviation or median (interquartile range), whereas categorical variables are expressed as numbers (%).

**Table 3.** **Results of stepwise linear regression analyses of patients with ADHF**.

Variables	Log amylase					Log lipase				
	B	SE	95% CI	Partial correlation coefficient	*p*	B	SE	95% CI	Partial correlation coefficient	*p*
			Lower					Lower		
			Upper					Upper		
AF	-	-	-	-	-	0.287	0.127	0.035	0.287	0.026
								0.538		
BUN	0.019	0.003	0.012	0.530	<0.001	-	-	-	-	-
			0.025							
Creatinine	-	-	-	-	-	0.236	0.062	0.112	0.236	0.042
								0.359		
Log BNP	-	-	-	-	-	−0.162	0.064	−0.289	−0.257	0.013
								−0.034		
Albumin	0.264	0.106	0.053	0.252	0.015	-	-	-	-	-
			0.475							
Uric acid	−0.079	0.021	−0.119	−0.371	<0.001	-	-	-	-	-
			−0.038							

Abbreviations: B, unstandardized coefficient; SE, standard error.

**Fig. 2.**
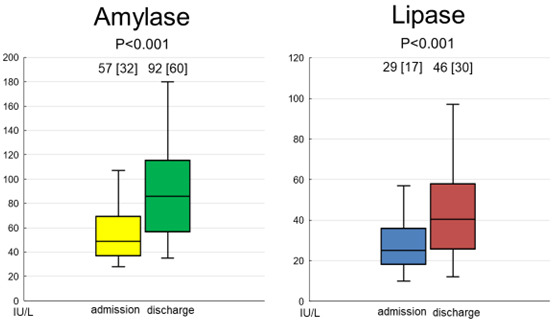
**Changes in the serum levels of amylase and lipase following the improvement of HF**. Both serum levels of amylase and lipase were significantly increased following the improvement of HF. Abbreviation: HF, heart failure.

In study 3, repeated measurements of serum amylase and lipase were performed in 90 patients with ADHF. Thirty patients who died, underwent surgical procedures, underwent dialysis during the period of hospitalization, were transferred to other hospitals for continued treatment, or lacked other follow-up data were excluded. Thus, only the data of 60 patients were assessed. The baseline demographics and medications of these patients are summarized in Table 4. The median and mean follow-up periods from admission to discharge were 16.0 and 25.0 d, respectively. All 60 patients survived and were discharged with improvements in HF. As shown in Fig. 2, the serum levels of amylase were significantly increased following the improvement of HF. Similarly, the serum levels of lipase were significantly increased following the improvement of HF. Changes in parameters other than amylase and lipase between admission and predischarge are summarized in Table 5. The body weight decreased significantly following the improvement of HF. The hemoglobin, serum albumin, and potassium levels increased, whereas the plasma BNP level decreased following the improvement of HF. In terms of the correlation between changes in amylase or lipase levels and changes in other parameters, a greater increase in amylase levels was associated with a greater reduction in body weight (correlation coefficient, –0.305; *p* = 0.033). In addition, an increase in lipase levels correlated significantly with the reductions in body weight (correlation coefficient, –0.338; *p* = 0.017) and serum creatinine levels (correlation coefficient, –0.266; *p* = 0.042). However, no significant correlations were found between amylase or lipase levels and other parameters.

**Table 4.** **Baseline characteristics of patients with ADHF who underwent repeated measurements of amylase and lipase levels**.

N = 60		
Age, years		70.5 ± 15.0
Male, n (%)		39.0 (65.0)
Body mass index, kg/m^2^		23.4 ± 5.4
Systolic blood pressure, mmHg		135.2 ± 32.6
Diastolic blood pressure, mmHg		83.3 ± 21.9
Heart rate, /min		97.1 ± 26.5
Left ventricular ejection fraction, %		41.0 ± 19.4
Nohria-Stevenson classification, n (%)		
	Subset B	49.0 (81.6)
	Subset C	9.0 (15.0)
	Subset L	2.0 (3.3)
Current smoking, n (%)		7.0 (11.0)
Habitual drinking, n (%)		11.0 (18.3)
Ischemic etiology, n (%)		27.0 (45.0)
AF, n (%)		31.0 (51.6)
History of heart failure, n (%)		35.0 (58.3)
Hemoglobin, g/dL		12.2 ± 2.1
Cholinesterase, IU/L		217.1 ± 65.1
Albumin, g/dL		3.1 ± 0.4
BUN, mg/dL		25.6 ± 14.4
Creatinine, mg/dL		1.26 ± 0.86
Uric acid, mg/dL		7.3 ± 2.0
Triglyceride, mg/dL		80.5 ± 44.8
LDL cholesterol, mg/dL		98.9 ± 30.7
HDL cholesterol, mg/dL		41.1 ± 11.4
Blood glucose, mg/dL		113.9 ± 51.8
HbA1c, %		6.5 ± 1.4
Sodium, mmol/L		139.3 ± 3.6
Potassium, mmol/L		4.1 ± 0.6
BNP, pg/mL		794 (924)
CRP, mg/dL		0.55 (1.97)
ACE-I/ARB, n (%)		33.0 (55.0)
β-blocker, n (%)		30.0 (50.0)
MR antagonists, n (%)		15.0 (25.0)
Diuretics, n (%)		33.0 (55.0)

Continuous variables are expressed as the mean ± standard deviation or as median (interquartile range), while categorical variables are expressed as numbers (%).

**Table 5.** **Changes in parameters other than amylase and lipase levels between admission and predischarge**.

N = 60	On admission	Predischarge	*p* value
Body weight, kg	66.1 ± 18.8	61.7 ± 17.3	<0.001
Hemoglobin, g/dL	12.2 ± 2.1	13.0 ± 2.4	<0.001
Albumin, g/dL	3.1 ± 0.4	3.5 ± 0.4	<0.001
BUN, mg/dL	25.6 ± 14.4	23.2 ± 11.5	0.176
Creatinine, mg/dL	1.26 ± 0.86	1.23 ± 0.89	0.588
Sodium, mmol/L	139.3 ± 3.6	138.5 ± 3.6	0.126
Potassium, mmol/L	4.1 ± 0.6	4.3 ± 0.4	0.022
BNP, pg/mL	794 (924)	491 (477)	<0.001

Continuous variables are expressed as the mean ± standard deviation or median (interquartile range).

## 4. Discussion

This study provides novel insights into the pathophysiology of HF, particularly regarding the damage to other organs such as the pancreas. First, patients with ADHF had significantly lower serum levels of amylase and lipase compared with the age-, sex-, and BMI-matched controls. Second, in patients with ADHF, lower BUN and serum albumin levels and higher serum uric acid levels were independent correlates of lower serum amylase levels. By contrast, the presence of AF, lower creatinine levels, and higher plasma BNP levels were the independent correlates of lower lipase levels. Third, significant increases in the serum levels of amylase and lipase were observed following the improvement of HF. Finally, these increases in amylase and lipase levels following the improvement of HF correlated with reductions in body weight. In addition, increases in lipase levels correlated with the reduction in serum creatine levels. These findings suggest that patients with ADHF are likely to have PEI, low serum levels of amylase are associated with poor nutritional status and worsening kidney function, and the serum levels of lipase are associated with poor nutritional status and congestion associated with HF. Furthermore, during ADHF treatment, PEI improved in proportion to the improvement of systemic congestion, as indicated by reductions in body weight and BNP levels. To our knowledge, no other studies have reported the association between serum levels of exocrine pancreatic enzymes and HF. Thus, this study is the first to demonstrate that serum exocrine pancreatic enzyme levels, as suggestive of PEI, may be associated with ADHF and related congestion.

The damage and/or dysfunction of other organs (i.e., lungs, kidneys, liver, and intestines) are commonly observed in patients with ADHF and are associated with an increased risk of mortality [19]. In ADHF, increases in hydrostatic left atrial pressure and mitral regurgitation lead to elevated pressure in the pulmonary capillaries, disrupting the balance of capillary Starling forces. These changes increase the transudation of fluid into the interstitium, causing pulmonary edema, increased lung stiffness, dyspnea, and respiratory failure [20]. In ADHF, renal insufficiency typically results from hypoperfusion of the kidney due to poor forward flow or excessive diuresis [21]. In addition, elevated central venous pressure may worsen renal function through several mechanisms, including pressure-induced reduction in renal blood flow, renal hypoxia, increased interstitial pressure, and interstitial fibrosis [22]. Hepatic dysfunction is closely associated with renal dysfunction in HF (i.e., cardio–renal–hepatic syndrome). The primary mechanisms underlying cardiac hepatopathy include reduced arterial perfusion, which is worsened by concomitant hypoxia, and passive congestion secondary to increased systemic venous pressure [23]. Systemic/venous congestion, sympathetic vasoconstriction, and low cardiac output contribute to decreased flow in the splanchnic microcirculation, increasing the risk of bowel ischemia. Ischemia causes epithelial cell dysfunction and the loss of intestinal barrier function, allowing lipopolysaccharides or endotoxins produced by gram-negative gut bacteria to enter the bloodstream. These substances trigger systemic inflammation and cytokine generation, disrupting cardiomyocyte function and energetics [24]. In addition, splanchnic congestion was commonly observed in cachectic patients, as evidenced by increased bowel wall thickness, which was associated with appetite loss, postprandial fullness, and inflammation [25].

Pancreatic lesions, ranging from subclinical hyperamylasemia to severe necrotizing pancreatitis, have been reported in several patients undergoing cardiac surgery [10]. One important risk factor for the development of pancreatic injury is ischemia associated with hypoperfusion [10, 26]. An acute increase in serum pancreatic enzyme levels occurs after exposure of the pancreas to ischemia/hypoperfusion, even for brief durations. Moreover, the percentage increase in the serum levels of pancreatic amylase and lipase above baseline values, an indicator of the degree of acinar cell injury, was significantly greater in patients with longer clamping times [10]. Thus, ischemia/hypoperfusion in the pancreas results in increased serum levels of pancreatic enzymes. In our study, only 21% of patients with potential hypoperfusion (i.e., Nohria-Stevenson classification C and L) were identified, which may contribute to our findings. Conversely, Vonglahn and Chobot [14] found that chronic passive congestion due to ADHF may lead to acinar cell atrophy and the disappearance of the prezymogen granules, both of which may result in decreased serum pancreatic exocrine enzyme levels. Furthermore, although the interval from the first episode of ADHF to death did not have an effect, a longer duration congestion associated with recent ADHF episodes was linked to more advanced changes in pancreatic tissues [14]. This suggests that congestion in ADHF can alter the pancreatic tissue morphology and possibly exocrine function. In study 1, we demonstrated that patients with ADHF had significantly lower serum amylase and lipase levels compared with the control group. These low serum levels are likely associated with the abovementioned changes in pancreatic tissues and PEI in association with congestion, as low serum pancreatic enzyme levels have been observed in patients with PEI [27, 28]. Additionally, low serum pancreatic enzyme levels can aid in screening for PEI [29]. Indeed, study 2 found correlations between lower lipase levels, the presence of AF, and higher BNP levels, all of which typically indicate greater congestion in patients with ADHF [30, 31]. These findings suggest that congestion associated with ADHF may contribute to decreased serum levels of pancreatic exocrine enzymes through the alteration of pancreatic tissues. On the contrary, the correlation between decreased levels of serum pancreatic exocrine enzymes and creatinine (related to muscle quantity) as well as BUN and albumin levels (which reflect nutritional status) suggests that a decrease in serum levels of pancreatic exocrine enzymes may impair digestion and absorption, leading to malnutrition. Similar findings were observed in a previous study among patients with chronic kidney disease [32]. Their study showed that serum amylase and lipase levels were directly correlated with serum creatinine levels, concluding that deficiencies in pancreatic exocrine functions may play important roles in the onset of chronic kidney disease-associated wasting syndrome [32]. This is further supported by the fact that an increased serum uric acid level, which is suggestive of kidney impairment, were associated with decreased serum amylase levels. Moreover, study 3 demonstrated that increases in serum amylase and lipase levels following the improvement of HF may indicate that atrophy of acinar cells and the disappearance of prezymogen granules in the pancreas are improved as the congestion associated with ADHF is alleviated. Thus, in patients experiencing congestion due to ADHF, the possibility of PEI should be evaluated, even in the absence of symptoms of pancreatic damage. Additionally, once patients experience malnutrition due to HF, independent of PEI, the condition may worsen as the pancreas requires optimal nutrition for enzyme synthesis [33], potentially leading to a vicious cycle. Therefore, supplementation of pancreatic enzymes, at least during the acute phase, is recommended to improve nutrition in patients with ADHF.

Our study has some limitations. First, this study was conducted at a single academic center and only included a Japanese patient population. Therefore, our findings should be examined in a larger and more diverse sample to enhance generalizability. Second, studies 1 and 2 were observational and cross-sectional in nature, which limits our ability to establish a cause-and-effect relationship between ADHF and PEI. A prospective cohort study would be more appropriate for investigating this causal link. Third, although no significant correlations were found between serum pancreatic enzyme levels and the use of diuretics and angiotensin-converting enzyme inhibitors, which are commonly used in patients with ADHF, these medications could still influence pancreatic function and enzyme levels. Additionally, other potential confounding factors may exist, which should be considered when exploring the relationships between ADHF and PEI. Fourth, in study 3, although we observed a significant increase in serum amylase and lipase levels, the findings were derived from an uncontrolled study. Thus, this introduces the possibility that changes in intravascular fluid volume and renal function may have influenced the serum levels of amylase and lipase. However, considering the correlation between reduced serum creatinine levels and increased serum lipase levels, along with the significant association between elevated serum amylase and lipase levels and reduced body weight, the latter may not be applicable in the present study. Finally, we did not directly assess the PEI. Future research should include specific assessments, such as a pancreatic function test or a fecal elastase-1 test. Thus, the present study serves as a proof-of-principle study, and further mechanistic studies are required, including direct assessments of exocrine pancreatic enzymes and investigation of the association between low serum levels of amylase and lipase and malnutrition and/or poor clinical outcomes.

## 5. Conclusions

In patients with ADHF, decreased serum amylase and lipase levels, suggestive of PEI, were associated with the severity of congestion. However, increases in the serum levels of these enzymes were observed following the improvement of ADHF. The correlation of decreased amylase levels with lower BUN and albumin levels and higher uric acid levels, and that between decreased lipase levels and decreased creatine levels suggest that PEI in patients with ADHF may be associated with malnutrition, possibly due to impaired digestion and absorption, and/or chronic kidney disease-associated wasting syndrome. As serum amylase and lipase levels are only suggestive and not definitive markers of PEI, a direct assessment of pancreatic function (e.g., fecal elastase-1) is necessary to confirm PEI. However, the present study suggested that the pancreas may be another target organ and can be injured by ADHF/congestion, leading to PEI. Further studies are needed to determine whether targeted therapy for this variable can improve the clinical course and prognosis in patients with ADHF, and how these values can guide the treatment of ADHF.

## Availability of Data and Materials

The data sets generated and analyzed during the current study are not publicly available but are available from the corresponding author on reasonable request.

## References

[1] Carr JG, Stevenson LW, Walden JA, Heber D (1989). Prevalence and hemodynamic correlates of malnutrition in severe congestive heart failure secondary to ischemic or idiopathic dilated cardiomyopathy. *The American Journal of Cardiology*.

[2] Rahman A, Jafry S, Jeejeebhoy K, Nagpal AD, Pisani B, Agarwala R (2016). Malnutrition and Cachexia in Heart Failure. *JPEN. Journal of Parenteral and Enteral Nutrition*.

[3] Narumi T, Arimoto T, Funayama A, Kadowaki S, Otaki Y, Nishiyama S (2013). Prognostic importance of objective nutritional indexes in patients with chronic heart failure. *Journal of Cardiology*.

[4] Iwakami N, Nagai T, Furukawa TA, Sugano Y, Honda S, Okada A (2017). Prognostic value of malnutrition assessed by Controlling Nutritional Status score for long-term mortality in patients with acute heart failure. *International Journal of Cardiology*.

[5] Shirakabe A, Hata N, Kobayashi N, Okazaki H, Matsushita M, Shibata Y (2018). The prognostic impact of malnutrition in patients with severely decompensated acute heart failure, as assessed using the Prognostic Nutritional Index (PNI) and Controlling Nutritional Status (CONUT) score. *Heart and Vessels*.

[6] Hirose S, Miyazaki S, Yatsu S, Sato A, Ishiwata S, Matsumoto H (2020). Impact of the Geriatric Nutritional Risk Index on In-hospital mortality and length of hospitalization in patients with acute decompensated heart failure with preserved or reduced ejection fraction. *Journal of Clinical Medicine*.

[7] Ronco C, Haapio M, House AA, Anavekar N, Bellomo R (2008). Cardiorenal syndrome. *Journal of the American College of Cardiology*.

[8] Canepa M, Straburzynska-Migaj E, Drozdz J, Fernandez-Vivancos C, Pinilla JMG, Nyolczas N (2018). Characteristics, treatments and 1-year prognosis of hospitalized and ambulatory heart failure patients with chronic obstructive pulmonary disease in the European Society of Cardiology Heart Failure Long-Term Registry. *European Journal of Heart Failure*.

[9] Nikolaou M, Parissis J, Yilmaz MB, Seronde MF, Kivikko M, Laribi S (2013). Liver function abnormalities, clinical profile, and outcome in acute decompensated heart failure. *European Heart Journal*.

[10] Gullo L, Cavicchi L, Tomassetti P, Spagnolo C, Freyrie A, D’Addato M (1996). Effects of ischemia on the human pancreas. *Gastroenterology*.

[11] Warshaw AL, O’Hara PJ (1978). Susceptibility of the pancreas to ischemic injury in shock. *Annals of Surgery*.

[12] Sakorafas GH, Tsiotos GG, Sarr MG (2000). Ischemia/Reperfusion-Induced pancreatitis. *Digestive Surgery*.

[13] Hackert T, Hartwig W, Fritz S, Schneider L, Strobel O, Werner J (2009). Ischemic acute pancreatitis: clinical features of 11 patients and review of the literature. *American Journal of Surgery*.

[14] Vonglahn WC, Chobot R (1925). The Histological Alterations of the Pancreas in Chronic Passive Congestion. *The American Journal of Pathology*.

[15] Sikkens ECM, Cahen DL, Koch AD, Braat H, Poley JW, Kuipers EJ (2013). The prevalence of fat-soluble vitamin deficiencies and a decreased bone mass in patients with chronic pancreatitis. *Pancreatology*.

[16] Domínguez-Muñoz JE (2011). Pancreatic exocrine insufficiency: diagnosis and treatment. *Journal of Gastroenterology and Hepatology*.

[17] Lesi C, Melzi D’Eril GV, Pavesi F, Scandellari A, Faccenda F, Grazia Casertano M (1985). Clinical significance of serum pancreatic enzymes in the quiescent phase of chronic pancreatitis. *Clinical Biochemistry*.

[18] McKee PA, Castelli WP, McNamara PM, Kannel WB (1971). The natural history of congestive heart failure: the Framingham study. *The New England Journal of Medicine*.

[19] Harjola VP, Mullens W, Banaszewski M, Bauersachs J, Brunner-La Rocca HP, Chioncel O (2017). Organ dysfunction, injury and failure in acute heart failure: from pathophysiology to diagnosis and management. A review on behalf of the Acute Heart Failure Committee of the Heart Failure Association (HFA) of the European Society of Cardiology (ESC). *European Journal of Heart Failure*.

[20] Ware LB, Matthay MA (2005). Clinical practice. Acute pulmonary edema. *The New England Journal of Medicine*.

[21] Guyton AC, Jones CE (1973). Central venous pressure: physiological significance and clinical implications. *American Heart Journal*.

[22] Legrand M, Mebazaa A, Ronco C, Januzzi JL (2014). When cardiac failure, kidney dysfunction, and kidney injury intersect in acute conditions: the case of cardiorenal syndrome. *Critical Care Medicine*.

[23] Møller S, Bernardi M (2013). Interactions of the heart and the liver. *European Heart Journal*.

[24] Verbrugge FH, Dupont M, Steels P, Grieten L, Malbrain M, Tang WHW (2013). Abdominal contributions to cardiorenal dysfunction in congestive heart failure. *Journal of the American College of Cardiology*.

[25] Valentova M, von Haehling S, Bauditz J, Doehner W, Ebner N, Bekfani T (2016). Intestinal congestion and right ventricular dysfunction: a link with appetite loss, inflammation, and cachexia in chronic heart failure. *European Heart Journal*.

[26] Fernández-del Castillo C, Harringer W, Warshaw AL, Vlahakes GJ, Koski G, Zaslavsky AM (1991). Risk factors for pancreatic cellular injury after cardiopulmonary bypass. *The New England Journal of Medicine*.

[27] Ammann RW, Akovbiantz A, Largiader F, Schueler G (1984). Course and outcome of chronic pancreatitis. Longitudinal study of a mixed medical-surgical series of 245 patients. *Gastroenterology*.

[28] Goldberg DM, Durie PR (1993). Biochemical tests in the diagnosis of chronic pancreatitis and in the evaluation of pancreatic insufficiency. *Clinical Biochemistry*.

[29] Kwon CI, Kim HJ, Korc P, Choi EK, McNulty GM, Easler JJ (2016). Can We Detect Chronic Pancreatitis With Low Serum Pancreatic Enzyme Levels?. *Pancreas*.

[30] Rossi A, Enriquez-Sarano M, Burnett JC, Lerman A, Abel MD, Seward JB (2000). Natriuretic peptide levels in atrial fibrillation: a prospective hormonal and Doppler-echocardiographic study. *Journal of the American College of Cardiology*.

[31] Maisel WH, Stevenson LW (2003). Atrial fibrillation in heart failure: epidemiology, pathophysiology, and rationale for therapy. *The American Journal of Cardiology*.

[32] Ozkok A, Elcioglu OC, Cukadar T, Bakan A, Sasak G, Atilgan KG (2013). Low serum pancreatic enzyme levels predict mortality and are associated with malnutrition-inflammation-atherosclerosis syndrome in patients with chronic kidney disease. *International Urology and Nephrology*.

[33] Cleghorn GJ, Erlich J, Bowling FG, Forrest Y, Greer R, Holt TL (1991). Exocrine pancreatic dysfunction in malnourished Australian aboriginal children. *The Medical Journal of Australia*.

